# Isolation, antibiogram and pathogenicity of *Salmonella* spp. recovered from slaughtered food animals in Nagpur region of Central India

**DOI:** 10.14202/vetworld.2016.176-181

**Published:** 2016-02-16

**Authors:** D. G. Kalambhe, N. N. Zade, S. P. Chaudhari, S. V. Shinde, W. Khan, A. R. Patil

**Affiliations:** Department of Veterinary Public Health, Nagpur Veterinary College, Nagpur, Maharashtra, India

**Keywords:** antibiogram, Congo red binding assay, food animals, hemolysis, *Salmonella* Typhimurium

## Abstract

**Aim:**

To determine the prevalence, antibiogram and pathogenicity of *Salmonella* spp. in the common food animals slaughtered for consumption purpose at government approved slaughter houses located in and around Nagpur region during a period of 2010-2012.

**Materials and Methods:**

A total of 400 samples comprising 50 each of blood and meat from each slaughtered male cattle, buffaloes, pigs and goats were collected. Isolation was done by pre-enrichment in buffered peptone water and enrichment in Rappaport-Vassiliadis broth with subsequent selective plating onto xylose lysine deoxycholate agar. Presumptive *Salmonella* colonies were biochemically confirmed and analyzed for pathogenicity by hemolysin production and Congo red dye binding assay (CRDA). An antibiotic sensitivity test was performed to assess the antibiotic resistance pattern of the isolates.

**Results:**

A total of 10 isolates of *Salmonella* spp. from meat (3 from cattle, 1 from buffaloes and 6 from pigs) with an overall prevalence of 5% among food animals was recorded. No isolation was reported from any blood samples. Pathogenicity assays revealed 100% and 80% positivity for CRDA and hemolytic activity, respectively. Antimicrobial sensitivity test showed multi-drug resistance. The overall resistance of 50% was noted for trimethoprim followed by ampicillin (20%). A maximum sensitivity (80%) was reported to gentamycin followed by 40% each to ampicillin and trimethoprim, 30% to amikacin and 10% to kanamycin.

**Conclusion:**

The presence of multidrug resistant and potentially pathogenic *Salmonella* spp. in slaughtered food animals in Nagpur region can be a matter of concern for public health.

## Introduction

*Salmonella* is a genus within the Enterobacteriaceae family distributed worldwide, can cause serious disease in both humans and animals. Their pathogenic potential and abilities to harbor and spread resistance pose tremendous medical, public health and economic problems affecting animals and humans [1]. Outbreaks in animals are a zoonotic threat, but due to limited surveillance performed in animals it is difficult to identify human outbreaks that corresponds closely to an outbreak in a single animal species [2].

Pathogenicity of *Salmonella* has been widely studied with the *in-vivo* method of production of enterotoxin in rabbit using rabbit illial loop test. However, this test has certain limitations, *viz*., use of live animals and animal ethical issues. Assay such as Congo red (CR) binding test could prove a good alternative to *in-vivo* tests.

Although there is a plethora of information regarding pathogenesis and molecular biology of *Salmonella* spp., there is a paucity of information concerning the prevalence and incidence that serotypes associated with foodborne disease. About 80% of typhoid fever cases accounts from Bangladesh, China, India, Indonesia, Laos, Nepal, Pakistan, or Vietnam infecting roughly 21.6 million people and kills about 200,000 people every year [3].

Due to the high significance of *Salmonella* pathogen from public health point of view, the present work was aimed to determine the prevalence, antibiogram and pathogenicity of *Salmonella* in common food animals slaughtered in and around Nagpur region.

## Materials and Methods

### Ethical approval

Sampling and experiments have been carried out as per the guidelines laid down by the Institutional Ethical Committee and in accordance with local laws and regulations.

### Collection, transportation and isolation of samples

A total of 400 samples comprising 50 each of blood and meat samples from 50 each slaughtered male cattle, buffaloes, pigs and goats were collected from the government approved slaughter houses in and around Nagpur region. The purpose of selecting these food animals was that they were commonly preferred for consumption in the region. Moreover, the study relating to the prevalence of *Salmonella* among these food animals intended for consumption purposes was highly lacking in the region. The meat (approximately 50 g) and blood samples (15 ml) were collected aseptically in sterile polyethylene sachet and in sterilized test tubes, respectively, and were transported to the laboratory by maintaining cold chain inside an insulated box containing ice packs. Samples were immediately processed for isolation on arrival to the laboratory.

**Plate-1:**
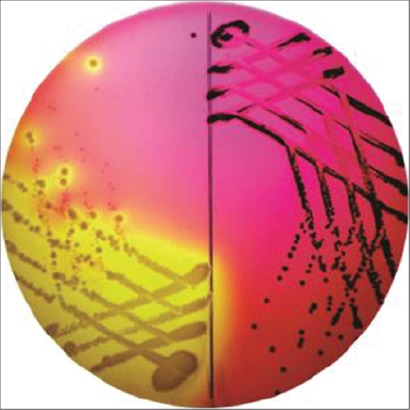
Characteristics colonies of *Salmonella* isolates on xylose lysine deoxycholate agar.

Meat samples each weighing 10 g were added with diluent (sterilized nine-salt solution [NSS]) and homogenized aseptically in stomacher blender (LabMed, UK), from this homogenized solution 1 ml was transferred to 9 ml buffered peptone water (BPW) similarly, the blood clots were inoculated into 9 ml BPW broth and incubated at 37°C for 24 h. After incubation, the inoculums from the respective pre-enriched broth were transferred to the second step enrichment media, i.e., Rappaport-Vassiliadis enrichment (RV) broth and further incubated at 44°C for 24 h. The enriched inoculums from RV broth were streaked onto xylose lysine deoxycholate (XLD) agar and incubated at 37°C for 24 h. Translucent colonies with typical black center were considered to be *Salmonella* (Plate-1). Each time along with the test samples standard *Salmonella* Typhimurium (MTCC-98) was processed as a reference positive culture and sterile NSS was used as a negative control.

The presumptive *Salmonella* colonies were subjected to Gram-staining which revealed the organisms as Gram-negative cocobacillary rods. Further, the Gram-negative organisms were subjected to biochemical characterization, sugar fermentation, *in-vitro* pathogenicity tests and antibiotic susceptibility.

**Plate-2:**
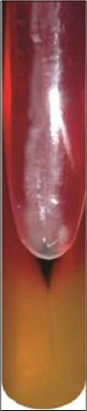
Typical patterm of *Salmonella* on triple sugar iron agar.

Isolates were biochemically characterized by urease, triple sugar iron (Plate-2), methyl red, Voges–Proskauer, indole, citrate, nitrate, malonate utilization tests and carbohydrates fermentation reaction using lactose, sucrose, salicin, adonitol, glucose, dulcitol, inositol, sorbitol sugars (Table-1).

**Table-1:** Biochemical characteristics of isolates.

Characteristics	Reaction
Gram’s-staining	Gram-negative, coccobacillary rods
Acid and gas from carbohydrates	
Lactose	−ve
Sucrose	−ve
Salicin	−ve
Adonitol	−ve
Glucose	+ve
Dulcitol	+ve
Inositol	±ve
Sorbitol	+ve
Methyl red	+ve
Voges–Proskauer test	−ve
Indole	−ve
Citrate	+ve
Nitrate	+ve
Urease	−ve
Malonate utilization	−ve
TSI	+ve (pink slope, yellow butt and H_2_S production indicated by blackening)

### Antibiotic sensitivity test

Antibiotic sensitivity of *Salmonella* isolates to various antibiotics and therapeutic agents *viz*., ampicillin (A) (10 mcg/unit), gentamicin (G) (5 mcg/unit), kanamycin (K) (30 mcg/unit), amikacin (Ak) (30 mcg/unit), and trimethoprim (Tr) (5 mcg/unit) was studied by agar disc diffusion method. Briefly, the overnight incubated cultures were spread evenly onto the brain heart infusion (BHI) agar plates by sterile swabs. The antibiotic disc was then placed with the help of sterile forceps onto the agar plates and incubated at 37°C for 24 h. The characterization of strains as sensitive, moderately sensitive and resistant was based on the size of zones of the inhibition around each disc according to manufactures instruction (Hi-media, Mumbai).

### *In-vitro* pathogenicity tests

Further pathogenicity was determined by applying *in-vitro* pathogenicity tests *viz*.; hemolysin production and CR binding assay.

### Hemolysis on sheep blood agar (SBA)

**Plate-3:**
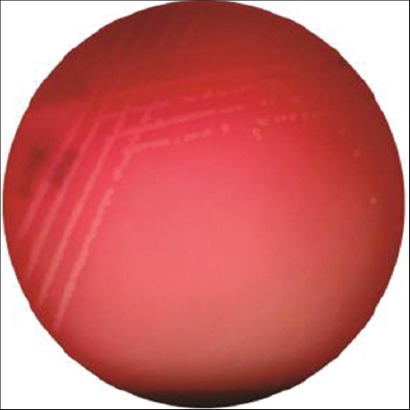
Hemolysin production of *Salmonella* isolate on sheep blood agar.

Detection of hemolysin production was conducted on 5% SBA. Defibrinated sheep blood was used for the preparation of 5% SBA plates. Freshly grown broth cultures were streaked onto the blood agar plates and incubated at 37°C for 24 h. Zone of hemolysis around the colonies was identified as α-hemolysis and accordingly the isolate was designated as pathogenic (Table-2 and Plate-3).

**Table-2:** Pathogenicity of the isolates.

Species	Cattle	Buffalo	Goat	Pig
Total positive samples				
Meat	03	01	00	06
Pathogenicity (%)				
Hemolysis	100	100	00	66.66
Cong red binding	100	100	00	100

### CR binding assay

**Plate-4:**
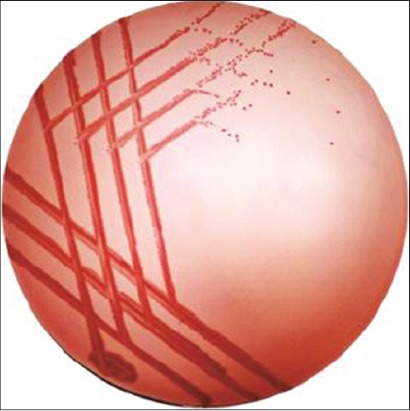
Typical (brick red) colonies Congo red agar.

The ability of the isolates to bind CR dye was evaluated by streaking freshly grown culture onto the CR BHI agar plates (0.003%) and incubating at 37°C for 24-48 h. The positive results were indicated by the formation of typical brick red colonies. The evaluation of the pathogenicity as +++, ++ and + was done based on intensity of brick red color ­development in colonies (Table-2 and Plate-4).

## Results

### Isolation, biochemical characteristics, and pathogenicity of the isolates

In the present investigation, a total of 400 samples (comprising 50 each of meat and blood) from respective slaughtered cattle male animals, buffalo, goat and pig (50 each) were screened for studying the prevalence, pathogenicity and antibiogram of *Salmonella* spp.

Of 400 samples, 10 meat (three beef, one buffalo beef, zero chevon and six pork) samples were assumed as *Salmonella* based on their colony characteristics and biochemical profile (Table-1 and Plates-1 and 2). However, none of the chevon and blood sample from any food animals was found to be positive for *Salmonella* spp. Cent percent beef and buffalo beef isolates turned positive for hemolysin production and CR binding assay, whereas 66.66% and 100% pork samples revealed positivity for hemolysin and CR binding assay (Tables-2 and 3 and Plates-2 and 3).

**Table-3:** Isolation of *Salmonella* from meat and blood.

Species	Cattle	Buffalo	Goat	Pig
Total samples				
Meat	50	50	50	50
Blood	50	50	50	50
Total positive samples				
Meat	03	01	00	06
Blood	00	00	00	00

### Antibiotic sensitivity patterns

Out of total 10 isolates screened for antimicrobial sensitivity test against five antibiotics, five isolates were found to be resistant for trimethoprim, whereas resistance against ampicillin was observed among two isolates registering resistance of 50% and 20%, respectively. The moderate sensitivity of 90% was reported for kanamycin followed by amikacin (70%), ampicillin (40%), gentamycin (20%), and trimethoprim (10%). A maximum sensitivity (80%) was reported to gentamycin followed by 40% each to ampicillin and trimethoprim, 30% to amikacin and 10% to kanamycin (Table-4).

**Table-4:** Antibiotic sensitivity patterns.

Antibiotics	Cattle (3 isolates)			Buffalo (1 isolates)			Pig (6 isolates)			Total isolates (10)		
			
	R	MS	S	R	MS	S	R	MS	S	R (%)	MS (%)	S (%)
A	00	02	01	00	01	00	02	01	03	02 (20)	04 (40)	04 (40)
G	00	01	02	00	00	00	00	01	06	00	02 (20)	08 (80)
K	00	03	00	00	01	00	00	05	01	00	09 (90)	01 (10)
Ak	00	03	00	00	00	01	00	04	02	00	07 (70)	03 (30)
Tr	02	00	01	00	01	00	03	00	03	05 (50)	01 (10)	04 (40)

R=Resistant, MS=Moderately sensitive, S=Sensitive, A=Ampicillin, G=Gentamicin, K=Kanamycin, Ak=Amikacin, Tr=Trimethoprim

## Discussion

The results of the present investigation, i.e., 6% *Salmonella* prevalence in beef and 2% in carabeef were comparable to the findings of 7.7% from minced beef from supermarkets in Addis Ababa, 9% prevalence from raw beef samples reported from Ethiopia [4] and 4.2% prevalence in slaughtered cattle reported in Ethiopia [5]. In a study conducted at Washington *Salmonella*, contamination rate in beef cattle carcasses was in the range of 0.2-21.5% [6]. However, an overall 6% *Salmonella* prevalence among beef samples in the current work was higher than those reported as 4% (02/50) in fecal samples from diarrheic young animal, from Telangana, Chennai, Maharashtra, Goa, Uttar Pradesh, and Rajasthan. The workers attributed the reason for lower prevalence in young animals and absences of *Salmonella* in bovine and equine samples to the limited number of samples included in the study [7]. Obtained higher prevalence in the present study could be due to the actual prevalence of *Salmonella* in cattle itself or it may be due to the unhygienic conditions in the slaughter house while dressing and handling the carcasses leading to the cross-contamination of the meat samples.

The prevalence of 2% in buffaloes beef is on the lower side when compared with the findings of the prevalence of *Salmonella* as 13.5% (5/37) in various meat samples including buffalo beef from the local meat market of Kathmandu [8]. The study from Anand, (Gujarat) India recorded (10.66%) prevalence of *Salmonella* spp. in raw buffalo meat and offals *viz*. liver, lung, muscle, intestine, and ground beef [9]. However, other worker isolated 8% (4/50) *Salmonella* organisms from buffalo meat samples from Laos (Japan) [10]. The results of the current work, however are comparable with the findings of (4.0%) prevalence in beef samples in Bareilly city, Izatnagar, India using cultural and polymerase chain reaction method [11].

Due to paucity of work done particularly in buffaloes and carabeef in the region, it is bit difficult to compare our results with the other works on the prevalence of *Salmonella* in buffaloes from the same region. However, when compared with work on raw meat conducted by Maharjan *et al*. and Kshirsagar *et al*. [8,9] who recorded 13.5% and 8% prevalence, respectively, the lower prevalence rates in the present investigation might be attributed to the actual less occurrence of the pathogen in the host [12]. The reason for the prevalence obtained in the present work could be attributed to the fact that transportation of the samples for 24 h might have despaired cold chain resulting into overgrowth of contaminants with suppression of *Salmonella*.

Although none of the samples (chevon or blood) were positive for *Salmonella*, in this study. This result is, however, in accordance with the study conducted to assess the microbiological quality and prevalence of *Salmonella* spp. in goat carcasses slaughtered at retail shops of Parbhani city, Maharashtra where zero prevalence of *Salmonella* in goat samples was recorded [13]. A study from Anand, Gujarat recorded the incidence of *Salmonella* in chevon as (3.57%) [14]. Results of present finding also goes in parallel with the finding from Mumbai wherein workers reported the absence of *Salmonella* in mutton samples [15]. Another study from Jammu revealed 3.12% prevalence of *Salmonella* spp. in chevon using standard plate count [16]. Among the findings of the other workers 3% *Salmonella* prevalence was reported in goats slaughtered at Ethiopia [17], 3.3% (1/31) prevalence was reported in chevon samples of local meat market in Kathmandu, Nepal [8]. Further, a study at Washington has reported the prevalence of *Salmonella* in the range of 1-18.8% in goats [6].

The observations of present study differ from the works done in the other parts of India wherein, 17.6% prevalence of *Salmonella* was recorded in goats slaughtered at Bareilly (North India) and 38.33% *Salmonella* in chevon at Wardha district of Maharashtra, respectively [18,19].

The differences in the reported prevalence could be associated with the sampling procedures, type of sample, transportation of samples, isolation techniques or the actual difference in the occurrence and distribution of *Salmonella* in the study population itself.

The report of 12% prevalence of *Salmonella* in pork in present study is higher compared to the 8.6% prevalence of *Salmonella* in pigs in the Tarai region of Uttarakhand, India [20] and the 8% (6/75) obtained prevalence of *Salmonella* in the pork samples collected from the markets of Nigeria [21]. However, the results are comparable with 16.4% (9/55) positivity for *Salmonella* in 55 pork samples in Ethiopia [22].

The variation could be attributed to the differences in the geographic location and the sanitary conditions of the slaughterhouses from where the samples are collected.

This can also be explained on the fact that these studies are based on the fecal carriage and rectum/intestinal contents harboring pathogen. There was a general observation that accidental rupture of rectum or intestine, entry of pathogen may lead to contamination of carcass [23] and accidental rupture of intestine during slaughtering is major cause for carcass contamination, as an experiment on use of rectal bag at abattoir were found to reduce the bacterial load of *Yersinia enterocolitica* to 2% [24]. The present investigation confirms reservoir status of the pigs as compared to other food animals in this geographical area. Further, the observation also alarms public health significance of pork with respect to *Salmonella* in this region.

An overall prevalence of *Salmonella* in 6% food animals in current study is comparable with the findings of 5% (15/300) *Salmonella* positive samples (pork, chicken, chevon, beef, carabeef and mutton) from the municipal slaughter houses and the retail meat shops from Hyderabad Karnataka region of Karnataka state, India [25]. Thus, the findings of present work highlight the contamination of the meat with *Salmonella*, alarming the public health risk thus necessitate the strict measures for the proper maintenance of slaughterhouses in the region.

In this study, an attempt was made to determine the pathogenicity of obtained *Salmonella isolates* by CR binding assay and hemolysin assays which yielded results in acceptance with [26-28]. Findings on the hemolytic strains of *Salmonella* observed is in agreement with a study of hemolysin pattern of *Salmonella gallinarum* in poultry reporting multiplicity in the hemolysin production among the pathogenic strain [26]. The observation of 80% hemolytic isolates in the present study is in complete agreement with the findings of a study which employed CR binding test and hemolysin production test to determine virulence of *Salmonella* Typhimurium isolates from calves and human samples and observed 81.81% hemolytic strains from calves, however, it is in partial agreement with cent percent CR binding isolates in present study against the 75% reported in the above-mentioned study [26].

The observation of antibiogram in this study could be compared with previous study conducted in this region on foods of animal origin where workers reported 80% sensitivity to gentamycin [29]. The sensitivity in the findings can be attributed to the similar geographical area of study wherein perpetuation of the strain of pathogen of same clonal origin cannot be denied [29]. The results of the present study differs from the reports obtained from Bareilly India, wherein, workers observed most strains were resistant to streptomycin (84.8%) followed by kanamycin (58.7%), gentamicin (52.2%), ampicillin (50%), and oxytetracycline (50%). Few strains were resistant to cefotaxime (2.2%), amoxicillin (2.2%) and newer fluoroquinolones (6.5%) [30]. However, variation among the sensitivity for amikacin, ampicillin and trimethoprim the tune of 80% and 13.3% in the present study can be contributed by possible variation in serotype isolated in both the studies as the workers used MacConkey and *Salmonella*-Shigella agar for isolation while XLD was used in this study.

## Conclusion

An overall 5% prevalence of *Salmonella* among the food animals reported in present study was low, but the fact of the presence of multidrug resistant and potentially pathogenic *Salmonella* in food animals of Nagpur region cannot be ruled out which can be a matter of concern from public health point of view. Further, there is a need of conducting such study in the region on a regular basis to asses and compare the past and present status of this zoonotic pathogen in the foods of animal origin in the region in order secure animal and public health.

## Authors’ Contributions

DGK as a MVSc student conducted the work. NNZ and SPC guided and supervised the research work. WK edited and selected photographs needed for the work. SVS and ARP participated in analysis of samples. All authors read and approved the final manuscript.
